# The Fuchs corneal dystrophy-associated CTG repeat expansion in the *TCF4* gene affects transcription from its alternative promoters

**DOI:** 10.1038/s41598-020-75437-3

**Published:** 2020-10-28

**Authors:** Alex Sirp, Kristian Leite, Jürgen Tuvikene, Kaja Nurm, Mari Sepp, Tõnis Timmusk

**Affiliations:** 1 Department of Chemistry and Biotechnology, Tallinn University of Technology, Akadeemia tee 15, 12618 Tallinn, Estonia; 2 Protobios LLC, 12618 Tallinn, Estonia; 3 Present Address: Department of Neurology, University Medicine Göttingen, Waldweg 33, 37073 Göttingen, Germany; 4 Present Address: Center for Molecular Biology of Heidelberg University (ZMBH), 69120 Heidelberg, Germany

**Keywords:** Corneal diseases, Bipolar disorder, Mechanisms of disease, Transcriptional regulatory elements, Alternative splicing, Reporter genes, Reverse transcription polymerase chain reaction, Gene expression, Gene regulation, Microsatellite instability, RNA sequencing, Molecular neuroscience, Molecular biology, Neuroscience

## Abstract

The CTG trinucleotide repeat (TNR) expansion in *Transcription factor 4* (*TCF4)* intron 3 is the main cause of Fuchs’ endothelial corneal dystrophy (FECD) and may confer an increased risk of developing bipolar disorder (BD). Usage of alternative 5′ exons for transcribing the human *TCF4* gene results in numerous *TCF4* transcripts which encode for at least 18 N-terminally different protein isoforms that vary in their function and transactivation capability. Here we studied the *TCF4* region containing the CTG TNR and characterized the transcription initiation sites of the nearby downstream 5′ exons 4a, 4b and 4c. We demonstrate that these exons are linked to alternative promoters and show that the CTG TNR expansion decreases the activity of the nearby downstream *TCF4* promoters in primary cultured neurons. We confirm this finding using two RNA sequencing (RNA-seq) datasets of corneal endothelium from FECD patients with expanded CTG TNR in the TCF4 gene. Furthermore, we report an increase in the expression of various other TCF4 transcripts in FECD, possibly indicating a compensatory mechanism. We conclude that the CTG TNR affects *TCF4* expression in a transcript-specific manner both in neurons and in the cornea.

## Introduction

Transcription factor 4 (TCF4) is a basic helix-loop-helix transcription factor that plays a vital role in the development of the nervous and immune system^1–3^. *TCF4* is expressed in almost every tissue type in human^4,5^. In the brain *TCF4* expression peaks during late embryonic development and continues at a relatively high level during postnatal development^6,7^. Notably, transcription from *TCF4* gene can begin at multiple mutually exclusive 5′ exons leading to transcripts with varying composition of functional protein domains which modulate the ability of TCF4 to regulate transcription^4,8^.

TCF4 gene is implicated in susceptibility to schizophrenia, and mutations in TCF4 cause Pitt-Hopkins syndrome, a rare developmental disorder characterized by severe motor and mental retardation^6,9–11^. In addition, mutations in the gene regions included only in the longer isoforms of *TCF4* have been associated with intellectual disability^12^. Alterations in *TCF4* expression levels have been described in patients with depression^13^. An expansion of a CTG TNR, located in an alternative promoter region between *TCF4* exons 3 and 4 (known as CTG 18.1), upstream of *TCF4* 5′ exons 4a, 4b and 4c causes Fuchs' endothelial corneal dystrophy (FECD) and has been linked to Bipolar disorder (BD)^14,15^.

FECD is an ocular disorder associated with corneal edema and vision disruption having a varying prevalence between different populations affecting about 4% of people over 40 in the United States^16^. Out of the many genetic mutations associated with FECD the CTG TNR expansion in *TCF4* is thought to be one of the major factors in the development of the disease, as the presence of a *TCF4* allele with 50 or more CTG TNR-s confers an increased risk of developing the disease^17^. BD is a psychiatric disorder that affects up to 1% of the global population, causing severe mood alterations in affected patients^18^. It has been shown that a *TCF4* CTG TNR expansion of over 40 CTG TNR-s is frequent in a subset of patients with a severe type of BD and that the repeat expansion in *TCF4* may increase vulnerability to BD^15^.

Studies on the connection between the *TCF4* CTG TNR expansion and the mRNA levels of *TCF4* transcripts spanning the *TCF4* CTG TNR region have produced contradictory results. A study by Foja et al.^19^ reported that *TCF4* CTG TNR expansion is connected with a reduction in the levels of *TCF4* transcripts beginning in the proximity of the CTG TNR, whereas a study by Okumura et al.^20^ has reported that *TCF4* CTG TNR expansion is connected with an increase in overall *TCF4* levels and in the levels of *TCF4* transcripts beginning in proximity of the CTG TNR. Two other studies^21,22^ have reported no effect of the CTG TNR expansion on total *TCF4* mRNA levels.

It is currently unknown whether the *TCF4* CTG TNR expansion affects the levels of *TCF4* transcripts and total *TCF4* levels. Here, we hypothesized that the *TCF4* CTG TNR region contains functional promoters that regulate the transcription of nearby 5′ exons and that the activity of these promoters is altered by the length of *TCF4* CTG TNR expansion. For this, we first characterized the *TCF4* alternative promoter region containing the CTG TNR by identifying transcription start sites (TSS) and describing the splicing of nearby 5′ exons 4a, 4b, and 4c. We then used luciferase reporter assay to investigate whether the CTG TNR expansion could influence *TCF4* expression in primary neurons by affecting the ability of surrounding regulatory regions to promote transcription. Furthermore, RNA-seq data from corneal tissue of FECD patients with an expanded *TCF4* CTG TNR was analyzed to determine the expression levels of *TCF4* transcripts beginning both proximal and distal to the CTG TNR. Collectively, our results demonstrate that the CTG TNR expansion differentially modulates the activity of *TCF4* promoters.

## Results

### Transcription start and splice donor site usage of *TCF4* 5′ exons in vicinity of the CTG TNR

**Figure 1:**
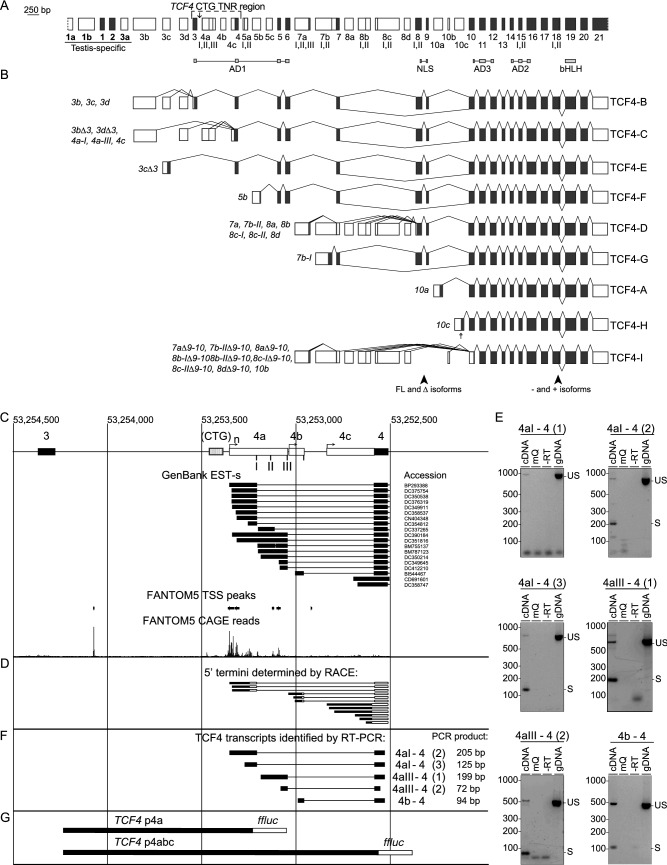
Transcription start sites, cloned insert sequences and analysis of splice sites in the *TCF4* CTG repeat-containing region in the adult human cerebellum. (**A**) A schematic of the TCF4 gene including alternative 5′ exons marked as white boxes and internal exons marked as black boxes drawn in scale. Functional protein domains have been marked under the exons and the *TCF4* CTG TNR region has been marked on top with an arrow displaying the repeat location in the intron between exons 3 and 4a. Alternative splice sites have been marked as roman numerals. Testis specific exons are underlined and marked bold. (**B**) Major alternative transcripts of the human *TCF4* gene. Coding regions are represented as black boxes and untranslated regions are shown as white boxes. Transcripts are named after the 5′ exon and the splice site. The names of protein isoforms encoded by the transcripts are shown on the right. Locations of alternative splicing that generates full-length (FL), Δ, − and + isoforms are shown at the bottom. A and B adapted from Sepp et al.^4^. (**C**) A schematic drawing of the TCF4 gene region proximal to the CTG TNR. Genomic coordinates are based on the GRCh37/hg19 genome build. Black boxes indicate internal exons, white boxes 5′ exons and striped open box marks the location of the CTG TNR. EST-s from GenBank are shown with accession numbers on the right. TSS peaks (FANTOM5 DPI peak, robust set) and CAGE reads (total counts of CAGE for reverse strand encoding for TCF4) from FANTOM5 project are visualized. (**D**) Transcripts identified by 5′ RACE are indicated—black boxes show sequenced components, white boxes unsequenced components. Transcription start regions of exon 4aI, exon 4b and exon 4c are indicated by arrows. (**E**) RT-PCR analysis of the splicing in the region. “US” indicates RT-PCR fragments amplified from gDNA or unspliced pre-mRNA. “S” denotes RT-PCR fragments from spliced mRNA. The numbers in brackets indicate different primer pairs used for PCR. Three different primer pairs were used to identify splice sites between exons 4a-I and 4, whereas two were used to identify splice sites between exons 4a-III and 4. (**F**) The structure of TCF4 transcripts identified by RT-PCR in D. (**G**) The cloned promoter regions are indicated with filled boxes and the firefly luciferase (ffLuc) reporter gene are indicated with an open box. *AD* activation domain, *NLS* nuclear localization domain, *bHLH* basic helix-loop-helix, *FL* full length, *Δ* lack of nuclear localization domain, *EST* expressed sequence tag, *TSS* transcription start site, *FANTOM5* functional annotation of the mammalian genome, *DPI* decomposition-based peak identification, *CAGE* cap analysis of gene expression.

The CTG TNR immediately precedes *TCF4* 5′ exons 4a, 4b and 4c, which are located between internal exons 3 and 4 (Fig. 1A). The major TCF4 transcripts transcribed from the alternative 5′ exons of *TCF4* in proximity of the CTG TNR are transcripts encoding for protein isoforms TCF4-B and TCF4-C (Fig. 1B). To characterize the transcription start sites (TSS-s) in this region we performed bioinformatical and 5′ RACE analysis. Analysis of GenBank data revealed that a total of 19 expressed sequence tags (EST-s) with 17 different TSS-s can be found between *TCF4* internal exons 3 and 4 with none beginning downstream of exon 3 and upstream of the CTG TNR (Fig. 1C). In addition, analysis of TSS peak data from the FANTOM5 (Functional Annotation of the Mammalian Genome) project revealed 6 TSS peaks near the CTG TNR: 1 TSS peak before the CTG TNR and 5 TSS peaks (of which 3 TSS peaks match with TSS-s from GenBank EST-s) downstream of the repeat (Fig. 1C). To validate the potential transcripts and TSS-s from our bioinformatical analysis, we performed 5′ RACE from adult human cerebellar RNA, as it exhibits high levels of *TCF4* expression^23^. Our 5′ RACE analysis revealed twelve TSS-s—three for exon 4a, three for exon 4b and six for exon 4c, distributed across a ~ 250 bp region (Fig. 1D). Out of the 12 TSS-s detected by 5′ RACE only 4 matched with the TSS-s from our bioinformatical analysis. Importantly, the CTG TNR was never present in the 5′ UTR of exon 4a, 4b and 4c transcripts since EST-s from GenBank and our 5′ RACE revealed no TSS directly upstream of the *TCF4* CTG TNR region (Fig. 1D, Supplementary Fig. S1). When considering previously published data about the TSS-s of *TCF4* exon 4a, 4b and 4c and data obtained from our 5′ RACE analysis, the promoters in the *TCF4* CTG TNR region appear to be dispersed promoters, which are defined as a type of promoter where transcription start sites are spread across a region of around 100 nucleotides^24–26^.

To study the usage of splice donor sites at the 5′ exons located near the CTG TNR, we amplified the fragments encompassing the splice sites from adult human cerebellar RNA using RT-PCR. We identified all previously described *TCF4* exon 4a and 4b splice sites^4^ in adult human cerebellum but could not detect mRNAs starting with exon 4a-II. Similar results were obtained in our previous study^4^, although one respective sequence is present in GenBank, suggesting that the levels of these *TCF4* transcripts are very low. In addition, we found that during splicing, donor site closest to the TSS is used. For instance, *TCF4* mRNAs initiated upstream of 4a-I splice site used donor site 4a-I exclusively and not downstream splice sites 4a-III or 4b (Fig. 1D–F). The RT-PCR analysis confirmed the absence of TSS-s directly upstream of CTG TNR (Fig. 1E). Collectively these results revealed that *TCF4* CTG TNR is not included in the 5′ UTR of exon 4a, 4b and 4c transcripts and instead locates in a dispersed promoter^24–26^ region characterized by spread TSS distribution.

### Activity of *TCF4* promoters immediately downstream of the CTG TNR decreases with increasing repeat length

**Figure 2:**
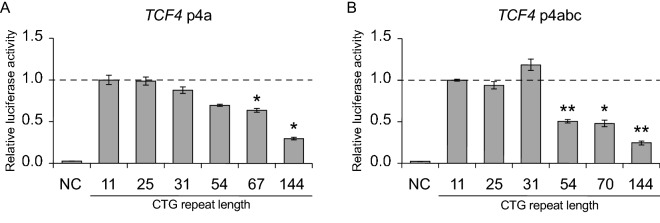
The *TCF4* CTG trinucleotide repeat region modulates the activity of proximal downstream *TCF4* promoters in a length dependent manner in neurons*.* (**A**,**B**) Promoter activities of *TCF4* p4a (**A**) and *TCF4* p4abc (**B**) constructs with different CTG repeat sizes (11, 25, 31, 54, 67 or 70, 144). Promoter activity was measured in rat cultured cortical neurons transfected with luciferase reporter constructs. Three independent experiments were performed for each promoter construct. A negative control (NC, promoterless pGL4.15 vector) is also shown. Statistical analysis was performed using one-way repeated-measures analysis of variance (ANOVA) with Greenhouse–Geisser correction followed by Dunnett’s post hoc test. Statistical significance is shown compared to the construct with 11 CTG repeats (*p < 0.05, **p < 0.01). Luciferase activity is shown relative to the luciferase signal obtained with the respective promoter construct with 11 CTG repeats.

We next determined whether the region surrounding the CTG TNR in *TCF4* intron 3 upstream of alternative 5′ exons contains functional promoters (Fig. 1C). For that, we analyzed two DNA fragments—a shorter sequence (*TCF4* p4a) spanning the *TCF4* CTG TNR region from just downstream of exon 3 into 5′ exon 4a and a longer sequence (*TCF4* p4abc) spanning the entire *TCF4* CTG TNR from just downstream of exon 3 to inside exon 4 (Fig. 1G). We cloned these fragments into pGL4.15[luc2P/Hygro] luciferase reporter vectors and transfected the vectors into rat primary cortical neurons. The expression of luciferase was increased by > 30-fold using reporter constructs containing either p4a or p4abc sequences when compared to a negative control construct without a promoter (Fig. 2A,B). These results indicate that p4a and p4abc sequences contain functional promoters.

To assess the effect of the CTG TNR length on the activity of *TCF4* p4a and p4abc promoter regions, we generated twelve luciferase reporter constructs where each construct contained either the *TCF4* p4a or p4abc promoter sequence combined with six different CTG TNR lengths (11, 25, 31, 54, 67/70, 144). The luciferase reporter assay revealed that an extended CTG TNR with a length of 54, 67/70 or 144 repeats significantly reduced the activity of the promoter for both *TCF4* p4a and p4abc (Fig. 2A,B). The presence of 144 repeats reduced the activity of p4a and p4abc by 70% (p = 0.0192) and 75% (p = 0.0095), respectively. These results demonstrate that the CTG TNR expansion interferes with transcription from the *TCF4* p4abc promoter region.

### The CTG TNR expansion in *TCF4* gene affects the transcription of *TCF4* alternative 5′ exons in FECD patients

**Figure 3:**
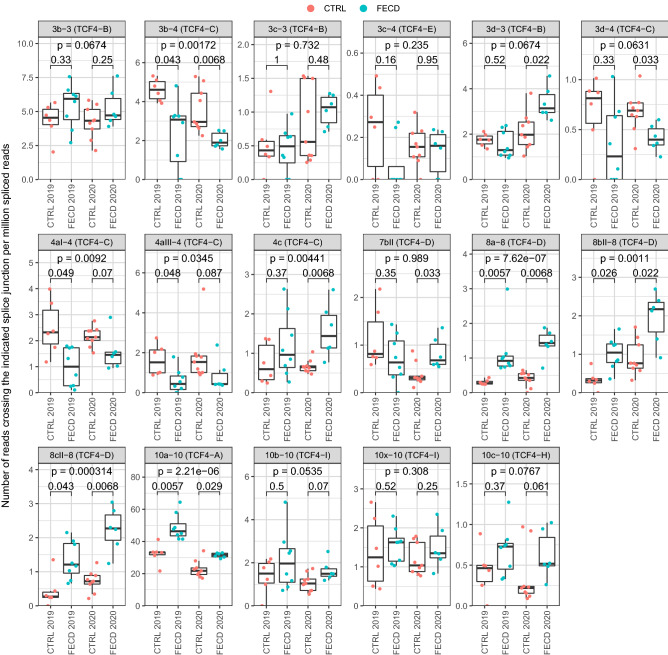
The expression of *TCF4* alternative transcripts containing 5′ exons spliced to exon 4 is decreased in the cornea of FECD patients*.* Two independent FECD RNA-seq datasets—by Nikitina et al.^27^ (named 2019 in the figure) and Chu et al.^28^ (named 2020 in the figure)—were used to analyze the expression levels of *TCF4* alternative 5′ exons in corneal endothelium. The expression levels of different TCF4 splicing events were quantified using the number of reads crossing the splice junction (shown above the graphs) normalized with the total number of spliced reads and multiplied by million. The TCF4 protein isoform encoded by the transcripts containing the indicated splicing event is shown in parentheses above the graphs. The data is visualized as box plots—the hinges show 25% and 75% quartiles, the horizontal line shows the median value, the upper whisker extends from the hinge to the largest value no further than 1.5 * inter-quartile range from the hinge, the lower whisker extends from the hinge to the smallest value at most 1.5 * inter-quartile range of the hinge. All data points are shown with dots. The 2019 dataset includes 6 controls and 8 FECD patients with an expanded *TCF4* CTG TNR, and the 2020 dataset includes 9 controls and 6 FECD patients with an expanded *TCF4* CTG TNR. 10 × –10 is a previously undescribed splicing event. Within-experiment statistical analysis between the CTRL and FECD patients (indicated groups) was done using Mann–Whitney U test, p-values were corrected for multiple testing using false discovery rate. Generalized linear model, followed by Wald test was used to determine statistical significance of the disease state combining data from both 2019 and 2020 experiments, p-values were corrected using FDR (p-value in the upper part of the panel).

To describe whether an increased CTG TNR affects the expression of different TCF4 transcripts, we performed a comprehensive analysis of two previously published RNA-seq datasets from the corneal endothelium of FECD patients with an expanded *TCF4* CTG TNR and control groups without the repeat expansion^27,28^. The 2019 dataset generated by Nikitina et al. includes 6 controls and 8 FECD patients with an expanded *TCF4* CTG TNR^27^, and the 2020 dataset by Chu et al. includes 9 controls and 6 FECD patients with an expanded *TCF4* CTG TNR^28^. First, we evaluated how the levels of transcripts beginning from the CTG TNR region change in FECD. The expression levels of exons 4aI and 4aIII showed a strong decrease in FECD patients, which is in agreement with our luciferase reporter assays in neurons (Fig. 3). In contrast, the levels of transcripts containing 5′ exon 4c were increased in FECD patients (Fig. 3).

Next, we investigated whether the CTG TNR affects the levels of TCF4 transcripts starting from far upstream of the CTG TNR (e.g. exons 3b, 3c, etc.). Our analysis revealed that FECD patients with an expanded CTG TNR displayed reduced levels of transcripts containing *TCF4* alternative 5′ exons 3b and 3d spliced directly to internal exon 4, just downstream of the repeat region, thus skipping internal exon 3 (Fig. 3). In contrast, the levels of transcripts containing these exons spliced to the internal exon 3 were either not changed (exons 3c and 3d) or were upregulated (exon 3b) in FECD. These results suggest that the CTG TNR affects both promoter activity and alternative splicing in transcripts starting from upstream of the repeat region (Fig. 3). Notably, we also found that FECD patients had increased levels of transcripts containing 5′ exons 8a, 8bII, 8cII and 10a, which are all located far downstream of the CTG TNR (Fig. 3).

**Figure 4:**
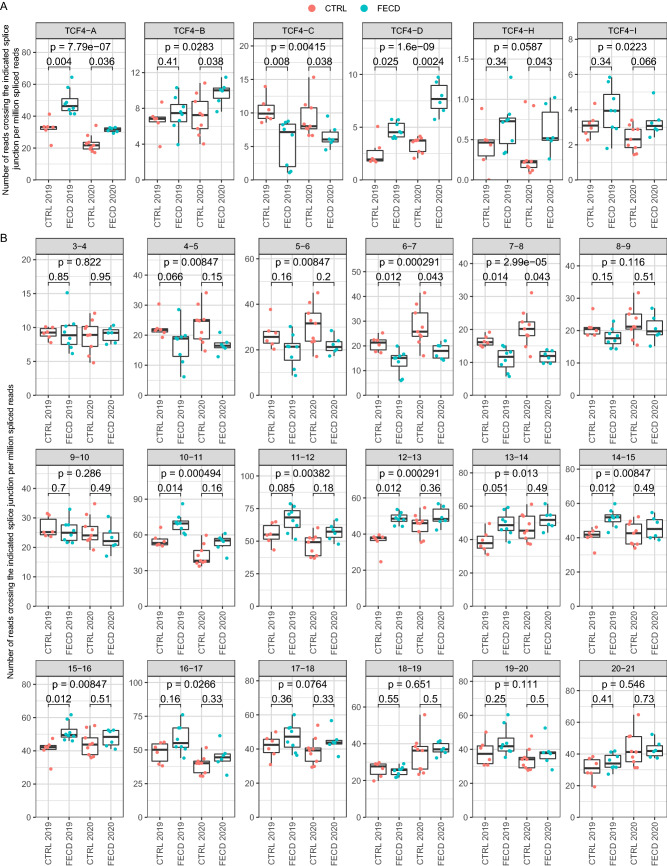
Expression of transcripts encoding TCF4-C isoform are downregulated, whereas transcripts encoding other TCF4 isoforms are upregulated in the cornea of FECD patients*.* Two independent FECD RNA-seq datasets—by Nikitina et al.^27^ (named 2019 in the figure) and Chu et al.^28^ (named 2020 in the figure)—were used to analyze the expression levels of *TCF4* exons in corneal endothelium. The expression levels of (**A**) TCF4 isoforms and (**B**) internal exons were quantified using the number of reads (**A**) from transcripts encoding for the respective isoform (shown above the graphs) and (**B**) crossing the indicated splice junction (shown above the graphs). Data was normalized with the total number of spliced reads and multiplied by million. For more details, see legend of Fig. 3.

**Figure 5:**
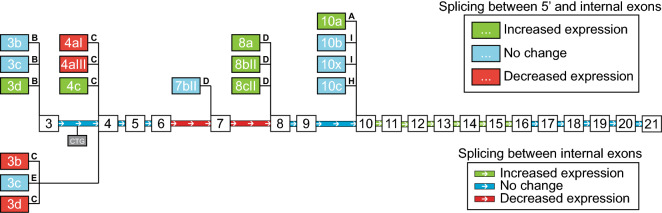
Summary of the expression levels of *TCF4* alternative 5′ and internal exons in FECD. Schematic depiction of the human *TCF4* gene structure and transcripts which displayed high expression levels in RNA-seq data analysis (see Figs. 4, 5). Internal exons are depicted as boxes (3–21) and 5′ exons are shown as columns on top or under the internal exons. Name of the respective *TCF4* exon is shown inside the box. Splicing of 5′ exons is shown by black lines which lead to the respective internal exon. Isoform encoded by the transcript is indicated as a single character (A, B, C, D, E, H or I) marked bold on the line next to the 5′ exon. 5′ exons marked green or red display significantly increased or decreased expression, respectively, in FECD, when spliced to the respective internal exon. Internal exons are connected by green, blue or red lines. A green or a red line between internal exons indicates a significant increase or decrease, respectively, in the expression level of transcripts containing splicing between these exons in FECD. A blue line or box indicates no change in the levels of transcripts containing splicing between these exons. The CTG TNR is marked between internal exons 3 and 4 as a grey box (CTG).

Different TCF4 transcripts encode for various TCF4 protein isoforms (Fig. 1B) that vary in their function and transactivation capability^4,8^. The major TCF4 transcripts comprising of 5′ exons 3b, 3c and 3d encode for isoform TCF4-B when spliced to internal exon 3; transcripts with exons 3b and 3d encode for isoform TCF4-C when spliced to exon 4; transcripts with exons 8a, 8bII and 8cII encode for isoform TCF4-D when spliced to exon 8; transcripts with exons 10a, 10b and 10c spliced to exon 10 encode for isoforms TCF4-A, TCF4-I and TCF4-H, respectively (Fig. 1B). Data from *TCF4* transcripts which encode the same protein isoform were combined to determine whether the levels of different TCF4 transcripts encoding specific TCF4 protein isoforms are changed in FECD. We found that the expression of transcripts encoding isoform TCF4-C decreased while the levels of isoforms TCF4-A, TCF4-B, TCF4-D and TCF4-H increased in FECD (Fig. 4). These observations were confirmed by analyzing *TCF4* transcripts by the expression levels of internal exons. Decreases in transcripts comprising of internal exons 6–8 were observed in FECD patients, which can be explained by reduced expression of isoform TCF4-C encoding mRNAs (Figs. 4, 5). FECD patients and the control group exhibited equal amounts of transcripts containing exons 8 and 9 (Fig. 4). The sudden elevation of transcripts comprising of exons 8 and 9 when compared to exons 4–8 in FECD patients accounts for the increase in the expression of isoform TCF4-D encoding mRNAs (Figs. 4, 5). An increase in the levels of transcripts containing exons 10–16 present in all *TCF4* transcripts is caused by the increase in the expression of TCF4-A, TCF4-B and TCF4-D mRNAs in FECD patients (Fig. 4). As a contradictory result we saw that transcripts containing exons 3 and 4 which should reflect the levels of isoform TCF4-B did not increase in FECD patients even though the levels of transcripts containing 5′ exons included in isoform TCF4-B did increase (Fig. 3).

Only the major transcripts/splicing events are reported in the Figs. 3, 4 and 5. The results of all studied *TCF4* exons/splicing events and isoforms can be found in Supplementary Fig. S2. In conclusion, the expression levels of *TCF4* transcripts were altered in FECD patients—the repeat expansion caused a reduction in transcripts starting immediately downstream of CTG TNR and transcripts containing 5′ exons spliced directly to exon 4, and an increase in transcripts encoded by distal 5′ exons located hundreds of kbp downstream of the repeat. The results of RNA-seq experiments have been summarized in Fig. 5.

## Discussion

Previous studies have indicated that the CTG TNR expansion in intron 3 of *TCF4* strongly increases the risk of developing FECD and also vulnerability and severity of BD^15,29^. The pathogenic mechanism of the *TCF4* CTG TNR and other TNR-s in general is still a major question. We investigated the hypothesis that the CTG TNR impacts the transcription of *TCF4* mRNAs initiated from nearby 5′ exons, leading to an imbalance of the levels of alternative TCF4 protein isoforms. We show for the first time that the expansion of the CTG TNR directly reduces the activity of the nearby downstream TCF4 promoters in a length dependent manner—longer, more expanded repeats reduce the activity of proximal downstream promoters. The lengths of the extended repeats that were studied fit into the pathogenic range for both bipolar disorder (> 40) and Fuchs’ dystrophy (> 50)^14,15^. Soliman et al. has shown that the severity of FECD correlates with the length of the CTG TNR in *TCF4* as patients with a CTG TNR expansion exhibited a more severe form of FECD, but the mechanism underlying this phenotype remains unknown^30^. It is important to note that our determination of TSS-s by 5′ RACE and reporter experiments were done using human cerebellar RNA and rat cultured cortical neurons, respectively. Therefore, it would be interesting to conduct similar experiments in human corneal endothelial cells. This would help to translate our findings between different cell types and further validate the effect of the CTG TNR expansion on transcription also in FECD patients.

Detailed analysis of previously published FECD RNA-seq datasets^27,28^ revealed that the levels of *TCF4* transcripts containing alternative 5′ exons 4aI and 4aIII were reduced in the corneal endothelial cells of FECD patients with an expanded CTG TNR. These results support our findings that the *TCF4* CTG TNR expansion reduces the activity of proximal downstream promoters linked to these 5′ exons also in human corneal endothelium. Interestingly, an increase in the levels of *TCF4* transcripts encoded by downstream alternative 5′ exons distal to the CTG TNR was also noted, which may indicate a compensatory mechanism to rescue the levels of TCF4 protein arising from the deficit of transcripts encoding TCF4-C. This compensation phenomenon needs to be considered when studying *TCF4* expression levels in FECD and other diseases connected with the *TCF4* intronic CTG TNR and could explain why different research groups have published contradictory results concerning changes in *TCF4* levels when studying FECD^19–22^. Our results indicate that the levels of *TCF4* transcripts change bidirectionally in response to an expanded CTG TNR—transcripts beginning near the repeat region decline just as Foja et al. reported^19^ while certain transcripts beginning downstream of the repeat region increase as reported here, and mask the decrease of long *TCF4* transcripts. As the expression of different *TCF4* transcripts decline and rise simultaneously the overall *TCF4* levels may not change significantly in FECD as has been reported by Mootha et al.^21^ and Ołdak et al.^22^. In contrast, Okumura et al.^20^ found an increase in total *TCF4* expression levels which is also evident in our RNA-seq analysis as we saw a slight rise in *TCF4* expression when measuring the expression of internal exons present in all TCF4 transcripts (exons 10–21). Our results illustrate the importance of the exact transcript measured when studying TCF4 expression levels. The original RNA-seq study by Chu and others concluded that the *TCF4* CTG TNR expansion increases the stability and thus the amount of expanded CTG repeat-including intronic RNAs in the corneal endothelium and causes comprehensive changes in splicing. No alterations in the overall expression of mature TCF4 mRNA was noted^28^. The study by Nikitina et al. was a data article and no conclusions were made^27^.

Interestingly, we also detected an increase in the expression of 5′ exons spliced to exon 3 encoding for TCF4-B in patients with FECD, showing that almost all the TCF4 promoters far upstream from the CTG TNR had increased activity due to the repeat expansion. However, the increase in upstream promoter activity did not reflect in the levels of transcripts containing exons 3 and 4. It is plausible that the CTG repeat expansion could regulate transcriptional elongation of RNA polymerase by slowing down the polymerase in the CTG TNR region^31^. This can cause an accumulation of RNA polymerases in the CTG TNR and dissociation of the polymerase, leading to a decrease of full-length TCF4 transcripts beginning from upstream of the CTG repeat. An increase in the expression of 5´ exons spliced to exon 3 may be due to preferential splicing of transcripts to the exon before the CTG TNR (exon 3), as changes in splicing have been described before in diseases associated with repeat expansion^31^.

We have previously shown that TCF4 protein isoforms can be divided into longer and shorter isoforms which vary in their function and transactivation capability^4,8^. Currently FECD research focuses mainly on the CTG TNR and missplicing of longer *TCF4* transcripts in FECD^17^, and little research has been done to analyze the expression of all the *TCF4* transcripts in FECD. Our detailed analysis provides new insight into FECD as we show that the CTG TNR directly modulates the expression of *TCF4* which may be among the underlying causes for the development of the disease. Since TCF4 mRNAs detected in the present study are expressed virtually in all tissues, with high levels in the fetal and postnatal brain^4^, there may also be a similar correlation between the CTG TNR length and the expression levels of *TCF4* transcripts in vivo in the brain which could predispose development of BD. However, it should be noted that the link between the CTG TNR expansion in *TCF4* and BD has only been shown once and has not been reported by newer studies.

Strong evidence has also been provided in support of a mechanism in which the toxic (CUG)_n_ TNR containing *TCF4 m*RNAs are the cause of Fuchs' corneal dystrophy^16,32,33^. According to this mechanism, the TNR-carrying RNAs cluster RNA binding proteins, interfering with the splicing of various mRNAs. Of note, antisense therapy using Fuchs' dystrophy ex vivo cell models leads to inhibition of RNA foci and mis-splicing in Fuchs' dystrophy^34,35^. Since the CTG TNR is located in the intron between exons 3 and 4, this repeat is not included in the fully mature *TCF4* mRNA^4^, but the CTG TNR is still included in the pre-mRNA of transcripts initiated at the upstream promoters (exon 1a, 1b, 3a, 3b, 3c, 3d promoters).

Repeat expansions have been associated with more than 40 diseases^29^ and unstable TNR-s may occur in both coding and noncoding regions, including promoters, introns and untranslated regions (UTR) of genes^36^. Among noncoding TNR-s, one of the most studied is the TNR repeat (CGG) located in the 5′ UTR of Fragile X Mental Retardation (FMR1) gene. This TNR causes hypermethylation and silencing or increases in the expression level of the gene, depending on TNR length^37^. TNR diseases with TNR in the promoter region of the affected gene have been less studied. Recently, an intronic polymorphic CGG repeat in a conserved alternative promoter of the AFF3 gene, an autosomal homolog of the X-linked AFF2/FMR2 gene, was shown to lead to hypermethylation of the promoter and transcriptional silencing of AFF3 expression in the brain^38^. However, the effect of TNR on promoter activity using transient expression analysis of promoters linked to TNR was not studied. Research on Friedreich ataxia, which is caused by an expansion of the intronic TNR (GAA) in the FXN gene, has revealed reduced expression of the gene in patient derived cell lines^39^. A hexamer repeat expansion (GGGGCC) located in the 5′ regulatory region of the C9ORF72 gene, causing hereditary amyotrophic lateral sclerosis, has been shown to reduce the ability of the surrounding region to promote the expression of a reporter protein in human kidney and neuroblastoma cell lines^40^. Overall, these results indicate that expansion of TNR can alter the expression of the nearby genes. This is in agreement with our results showing that the expression levels of different *TCF4* transcripts are altered in FECD due to the CTG TNR expansion.

Taken together, our results help to explain why previous research on the levels of *TCF4* transcripts in FECD has displayed varying results. Analyzing only total *TCF4* levels or levels of certain *TCF4* transcripts can produce misleading results due to the complexity of the *TCF4* gene and its regulation. The current study shows that the *TCF4* CTG trinucleotide repeat expansion modulates the activity of nearby *TCF4* promoters in a length dependent manner—an expanded CTG TNR causes reduction in promoter activity. Analysis of RNA-seq datasets revealed that the expression levels of the many *TCF4* transcripts are increased or decreased simultaneously in the cornea of FECD patients. Further work is needed to elucidate the exact mechanism how this repeat region affects TCF4 transcription and whether the changed TCF4 levels contribute to the development of FECD and BD.

## Methods

### Generation of DNA constructs

Human postmortem tissues were used to obtain DNA and RNA samples. All protocols using human tissue samples were approved by Tallinn Committee for Medical Studies, National Institute for Health Development (Permit Number 402). All experiments were performed in accordance with relevant guidelines and regulations.

*TCF4* gene fragments were screened from human DNA samples for the TCF4 CTG TNR length and fragments with the desired CTG TNR length were amplified by PCR from 20 ng of genomic DNA in a 20 μl mixture using 0.4 units of Phusion Hot Start II (Thermo Scientific) and primer p4a_p4abc_F paired with the p4abc_R or p4a_R primer (Supplementary Table S1) with a final concentration of 0.25 μM to amplify the longer (*TCF4* p4abc) and the shorter (*TCF4* p4a) sequence of the *TCF4* gene (Fig. 2). Following amplification, the PCR mixtures were incubated for 15 min at 72 °C with 1 unit of FirePol DNA polymerase (Solis BioDyne) for the synthesis of adenosine overhangs for cloning. The PCR products were first inserted into the pSTBlue-1 acceptor vector (Merck Millipore) and then to the pGL4.15[luc2P/Hygro] luciferase reporter vector (#E6701, Promega).

Promoter regions encompassing CTG TNR-s with five different lengths (11, 25, 31, 54 and 67 or 70 repeats) were acquired from human genomic DNA by PCR. A sixth synthetic DNA segment with 144 CTG repeats was ordered from GenScript. All the generated constructs were verified by sequencing as the length of TNR tended to be unstable in bacteria when producing plasmids (Supplementary Table S1).

### Luciferase reporter assay and neuron cultures

The protocols involving animals were approved by the ethics committee of animal experiments at Ministry of Agriculture of Estonia (Permit Number: 45). All experiments were performed in accordance with the relevant guidelines and regulations.

Prenatal rat cortical neurons were cultured as described previously^41^. Neurons grown 6 days in vitro were transfected with 180 ng firefly reporter construct and 20 ng pGL4.83[hRlucP/PGK1/Puro] as described previously^4^ for 4 h on a plate shaker using Lipofectamine 2000 (#11668019, Thermo Fisher Scientific) with a reagent to DNA ratio 3:1. Two days after transfection neurons were lysed in 50 μl Passive Lysis Buffer (Promega) and luciferase reporter assay was performed using the Dual-Glo Luciferase Assay System (Promega) according to manufacturer’s protocol. Luciferase signals were measured using the GENios Pro microtiter plate reader (Tecan). For analysis, the signals were first normalized to the signal of the Renilla luciferase and then normalized to the respective ratio in cells transfected with the 11 repeat CTG construct. One-way repeated-measures analysis of variance (ANOVA) with Greenhouse–Geisser correction followed by Dunnett’s post hoc test was used to determine the statistical significance compared to the luciferase signals from the 11 repeat CTG construct group.

### 5′ rapid amplification of DNA ends (5′ RACE) analysis and reverse transcription polymerase chain reaction (RT-PCR)

Total RNA from post-mortem adult human cerebellum was treated with Turbo DNase (Thermo Fisher Scientific) according to the supplier’s protocol. 5′ RACE analysis was carried out on human cerebellar RNA using the GeneRacer Kit (Thermo Fisher Scientific) according to manufacturer’s protocol with primers outlined in Supplementary Tables S1 and S2.

For RT-PCR, cDNA was synthesized from human cerebellar RNA using 100 units of SuperScript III reverse transcriptase (Thermo Fisher Scientific) with oligo(dT)_20_ and a random hexamer primer mixture (1:1 ratio, Microsynth) according to the manufacturer’s protocol. A negative control (− RT) was also included where Superscript III reverse transcriptase was not added. After cDNA synthesis, PCR was performed in 20 μl using 3 units of Hot FirePol (Solis Biodyne) and primers listed in Supplementary Table S1 with a final concentration of 0.25 μM. All the sense primers used for RT-PCR were combined with the antisense primer hTCF4_exon4_as2 except for sense primer hTCF4_4aIII_s (2) which was used together with the antisense primer hTCF4_exon4_as1.

### Bioinformatic analysis

Cap Analysis of Gene Expression (CAGE) data from the Functional Annotation of the Mammalian Genome project phase 5 (FANTOM5)^42^ was used to locate potential *TCF4* TSS-s. Both predicted TSS-s (FANTOM5 DPI, robust set) and total counts of CAGE reads for the reverse strand (encoding for TCF4) were visualized in UCSC Genome Browser together with the EST-s from GenBank (accessed at 10.07.2020) in the area surrounding the *TCF4* CTG TNR region (chr18:53,254,500–53,252,500, human GRCh37/hg19 assembly). The FANTOM5 data can be accessed at https://fantom.gsc.riken.jp/5/datahub/hg19/reads/ctssTotalCounts.rev.bw.

Raw RNA-seq data from corneal endothelium of FECD patients and controls (see Supplementary Table S3 for sample information) were obtained from Sequence Read Archive database (accession numbers PRJNA524323^27^ and SRP238609^28^) using prefetch tool (version 2.10.0) from the SRA toolkit. Reads in fastq format were extracted using fasterq-dump. Adapter and quality trimming was done using BBDuk (part of bbmap version 38.79) using the following parameters: ktrim = r k = 23 mink = 11 hdist = 1 tbo qtrim = lr trimq = 10 minlen = 100 (minlen = 85 for data from PRJNA524323). Reads were mapped to hg19 genome (primary assembly and annotation obtained from GENCODE, release 34, GRCh37) using STAR aligner (version 2.7.3a) with default parameters. To increase sensitivity for unannotated splice junctions, splice junctions obtained from the 1st pass were combined (per dataset) and filtered as follows: junctions on mitochondrial DNA and non-canonical intron motifs were removed; only junctions supported by at least 6 reads in the whole dataset were kept. The filtered junctions were added to the 2nd pass mapping using STAR. Intron-spanning reads were quantified using FeatureCounts (version 2.0.0) with the following parameters: -p -B -C -s 2 -J. To count reads from TCF4 extended exons (exons 4c and 7bII), reads crossing a region 2 bp 5′ from the internal exon (exon 4 and 7, respectively) were quantified using FeatureCounts and a custom-made saf file. Splice junctions in the TCF4 region showing less than 4 reads for the whole dataset were discarded, the rest of the splice junctions associated with TCF4 were manually curated and annotated according to Sepp et al.^4^. A custom R script was used to quantify the expression of different *TCF4* splice variants. Reads crossing the indicated splice junctions were normalized using the number of all splice-junction crossing reads in the respective samples. Then, data summed by the Exon column (see Supplementary Table S4) to obtain expression levels of splice junctions for TCF4 5′ exons. Next, data was aggregated by the Isoform column (see Supplementary Table S4) to obtain expression levels of spliced reads of *TCF4* internal exons and transcripts encoding different TCF4 protein isoforms. The annotated splice junction table for quantifying different TCF4 splice sites and transcripts encoding different isoforms is shown in Supplementary Table S4. The results were visualized using ggplot2 package (version 3.3.1) in R (version 4.0.1). Statistical analysis of the RNA-seq data was carried out in R as follows. To determine statistical significance between control and FECD patients within an experiment, non-parametric Mann–Whitney U-test was performed, p-values were corrected for multiple comparisons within experiment (per figure) using false discovery rate (FDR). To determine general statistical significance of the disease state for combined data of the two experiments, normalized data was transformed by adding 0.01, followed by fitting generalized linear model with Gamma distribution using Experiment + Disease + Experiment:Disease as the model. p-value for the disease state was obtained using Wald test and corrected for multiple comparisons using FDR (per figure).

## Supplementary information

Supplementary Information 1.

Supplementary Table S4.
