# Supplementary material for: The Fuchs corneal dystrophy-associated CTG repeat expansion in the TCF4 gene affects transcription from its alternative promoters
Source: Sci Rep. 2020 Oct 28;10:18424. doi: 10.1038/s41598-020-75437-3 (PMC7595208; doi:10.1038/s41598-020-75437-3)
Supplement: Supplementary file 1 — Supplementary Information 1. [file 41598_2020_75437_MOESM1_ESM.pdf]

The Fuchs corneal dystrophy-associated CTG repeat expansion in the *TCF4* gene affects transcription from its alternative promoters

Alex Sirp, Kristian Leite , Jürgen Tuvikene, Kaja Nurm, Mari Sepp, Tõnis Timmusk

```
   1  ACGTCCCCACCCCCAACCCCCCAGTCTCCAAAAATCCGATTGTGTTTTCTCCAAGGATTGGGACTGGATTTTCTGATTGCGGTTATTTCCATGTTTCTAG
                                                 Exon 3                                Splice site
 101  GTTTGTGTGATTTTGCTAAAATGCATCACCAACAGCGAATGGCTGCCTTAGGGACGGACAAAGAGCTGAGTGATTTACTGGATTTCAGTGCGGTAAGAAA

 201  GAACGGTGGAAACTAACAACAGCTGTGAAAAAAACAAAACAAAAACCCAAACACTTCAGCTAGAAACCAGTAGGAATCTAAAGGACAGTAATAATTTTTA
                                                               p4a_p4abc_F
 301  ATTGGCTGAATCCTTGGTAAATATGAAGGTCTTTTTGACAAGTTTTTAACTATAATTTTGTGGTGTGATGGAAGATTCAGGCTTTTTTTTTTTTTTGAGT

 401  TTTATTACTGGCCTTCAATTCCCTACCCACTGATTACCCCAAATAATGGAATCTCACCCCAGTGGAAAGCAAAAATAGACACCCCTAAAACTAAACCACC

 501  CCTAAAACTTGGCCATGTCTGAACACTGAGACTACTAATACTTTGCACACTACTCTTCGTTTTATTTATTGTTTTTGGAAATGGAAAATAGAAAATAGGA

 601  GACCCAGTTGTCTCTTTAAAGTTTTAAGCTAATGATGCTTTGGATTGGTAGGACCTGTTCCTTACATCTTACCTCCTAGTTACATCTTTTCCTAGGATTC

 701  TTAAAACTAGTATGGATATGCTGAGCATACATTCTTTAGAACCTTTTGGACTGTTTTGGTAAATTTCGTAGTCGTAGGATCAGCACAAAGCGGAACTTGA

 801  CACACTTGTGGAGTTTTACGGCTGTACTTGGTCCTTCTCCATCCCTTTGCTTCCTTTTCCTAAACCAAGTCCCAGACATGTCAGGAGAATGAATTCATTT

 901  TTAATGCCAGATGAGTTTGGTGTAAGATGCATTTGTAAAGCAAAATAAAAAGAATCCACAAAACACACAAATAAAATCCAAACCGCCTTCCAAGTGGGGC
                                       CTG trinucleotide repeat
1001  TCTTTCATGCTGCTGCTGCTGCTGCTGCTGCTGCTGCTGCTGCTGCTGCTGCTGCTGCTGCTGCTGCTGCTGCTGCTGCTGCTCCTCCTCCTCCTCCTCC
                         2            11
1101  TTCTCCTCCTCCTCCTCCTCTTCTAGACCTTCTTTTGGAGAAATGGCTTTCGGAAGTTTTGCCAGGAAACGTAGCCCTAGGCAGGCAGCTTTGCAGCCCC
                                                                 Splice site
1201  CTTTCTGCTTGTTGCACTTTCTCCATTCGTTCCTTTGCTTTTTGCAGGCTCTGACTCAGGGAAGGTGTGCATTATCCACTAGATACGTCGAAGAAGAGGG
                                                          p4a_R
                                          Splice site
1301  AAACCAATTAGGGTCGAAATAAATGCTGGAGAGAGAGGGAGTGAAAGAGAGAGTGAGAGTGAGAGAGAGAGAGAGTCTTGCTTCAAATTGCTCTCCTGTT
                            Splice site
                                     1                              1      1
1401  AGAGACGAATGAGAATTTAGTGCAGGTGGCACTTTTATTTTTATTTGGGTTCACATATGACAGGCAAATCCTATACGAGATGGAAATGGACATTGCCAC
              Splice site
1501  GTTTATGGCCAAGGTTTTCAATATAAAACAAAACAACTTTTTTCTTCTCCTTGGTGAAACTAGTGTTTTTCTAGAGAGGCTGCTGGCCTCCAACCTGAAT
                                      3           1                             3
1601  CTTGATAACATTATGGGGACTGTGTTTGTTCCAAATGTAGCAGTAGTACTGCTTGGCCATCTAATGAACCTGAGGAAAAAGAAAGAACAGAGTGATAATG
                                                                                          1
1701  GGGGCTGGGGTGGGATCTGTAATGTTGTTTCTCTTTTAGTTTTAAGTTGGATGGTGATGTATTTTACTAAATAAACCCTTAGCATAAACTCTAAGCTGTT
                1                                                                             Exon 4
1801  TGGTAACAGTATGAAAGATCTTTGAGGAGCTCTGAAGGCACAAGTGTCTTCTTTTCAACTGTAATATTTCTTTGTTTCTTTTAGATGTTTTCACCTCCTG
                                                                                     p4abc_R
                                                         Splice site
1901  TGAGCAGTGGGAAAAATGGACCAACTTCTTTGGCAAGTGGACATTTTACTGGCTCAAGTATGTAAATTCTTTTCTTAGTGTTAAGTTAAATGTTCTGGGC

2001  TGTTTATTTACTCAATTGTATGTTTTTGGCTCTGTTGAAGAGTTCTGAAAAAGTCATTGAATTTTAGCCTGTAATCATACGGTTATTGCATTACCCATCA
```

Supplementary Figure S1. Genomic sequence surrounding the *TCF4* CTG repeat region. The genomic area surrounding the CTG repeat extracted from the GRCh37/hg19 genome version. The bold numbers indicate 5’ RACE expressed sequence tags found in current study. TCF4 internal exons 3 and 4 are marked as a bolded and shaded sequence, and splice sites are marked with a bent line. Forward and reverse primers used for cloning the promoter constructs are underlined. Open box marks the CTG TNR.

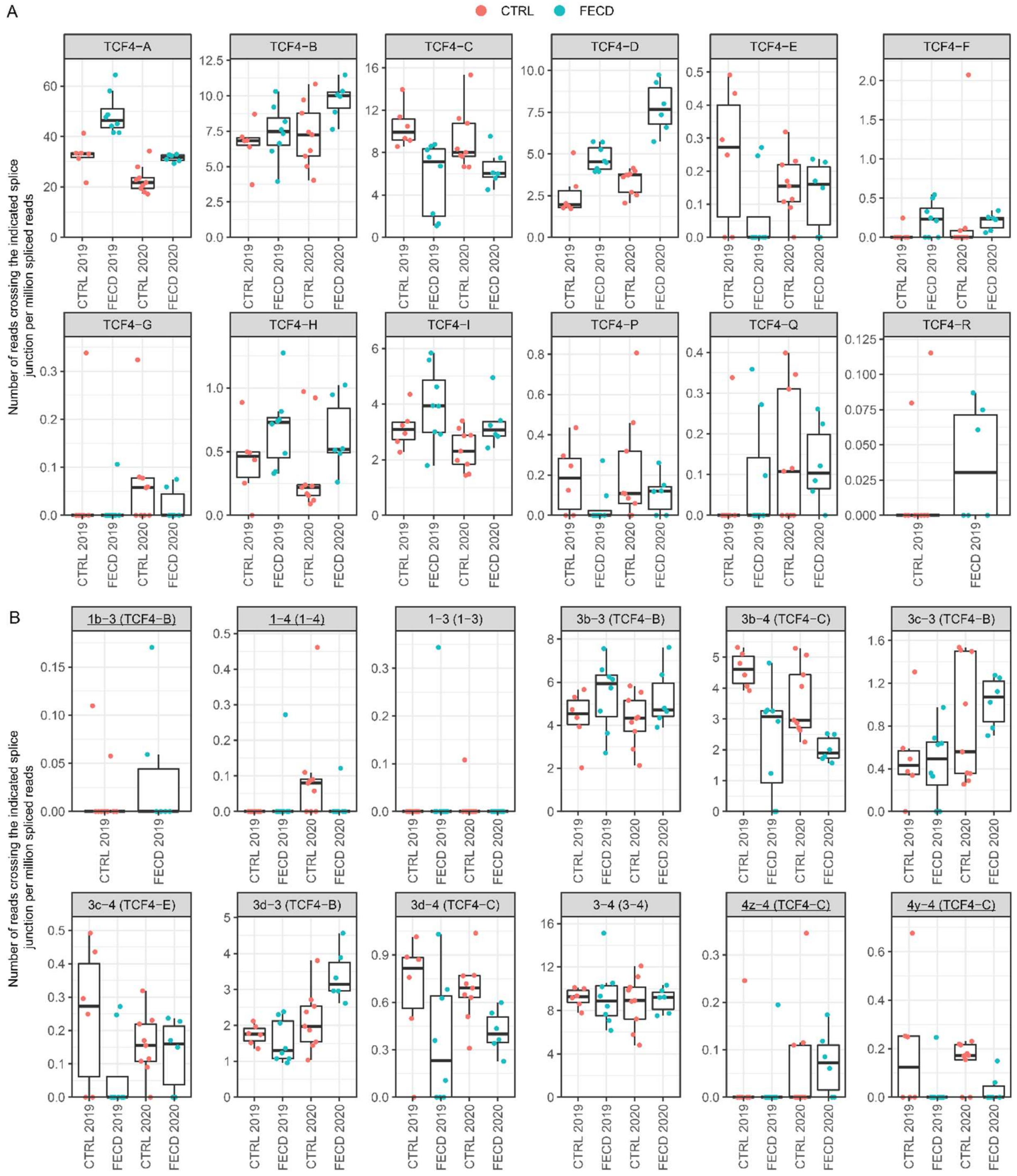

Figure continues on the next page.

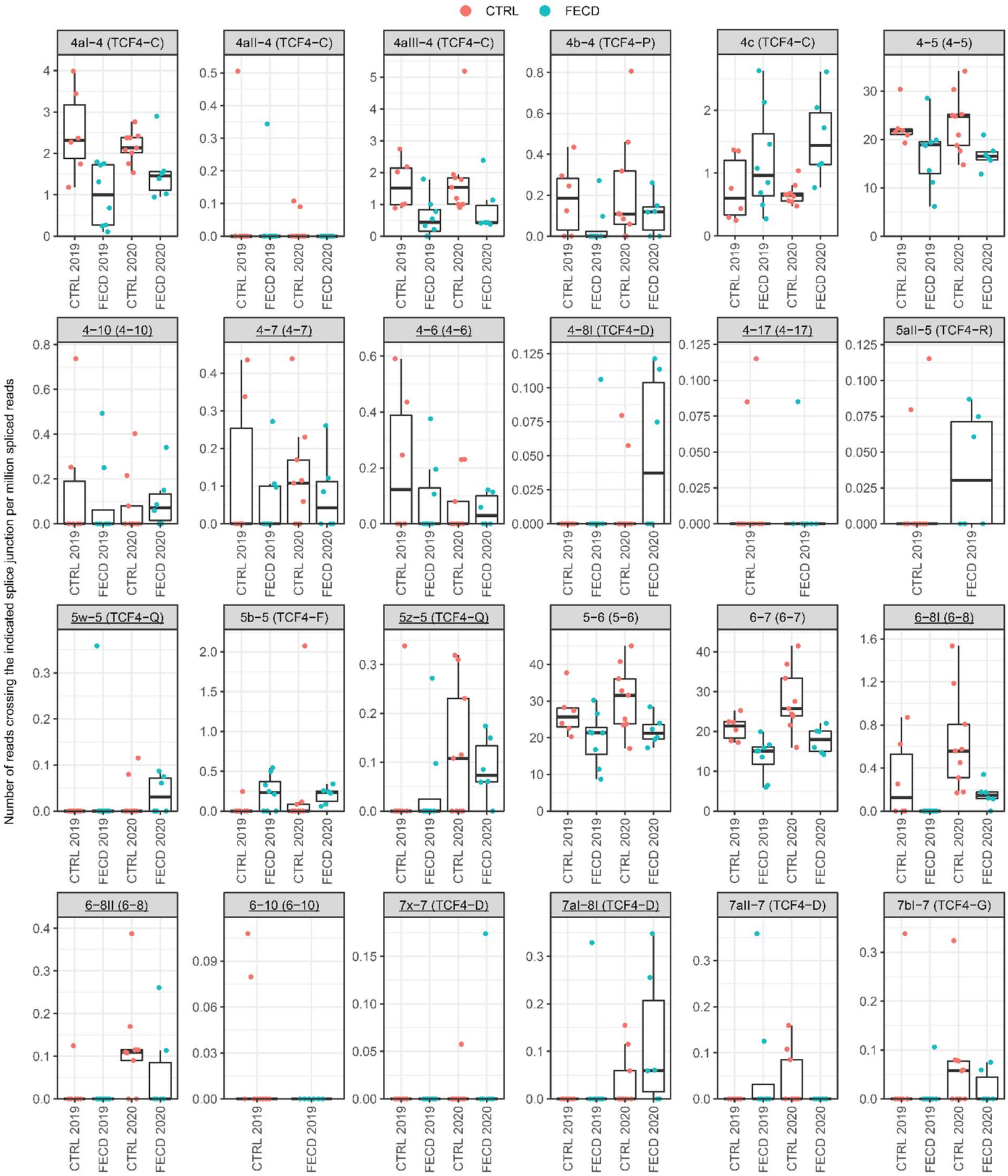

Figure continues on the next page.

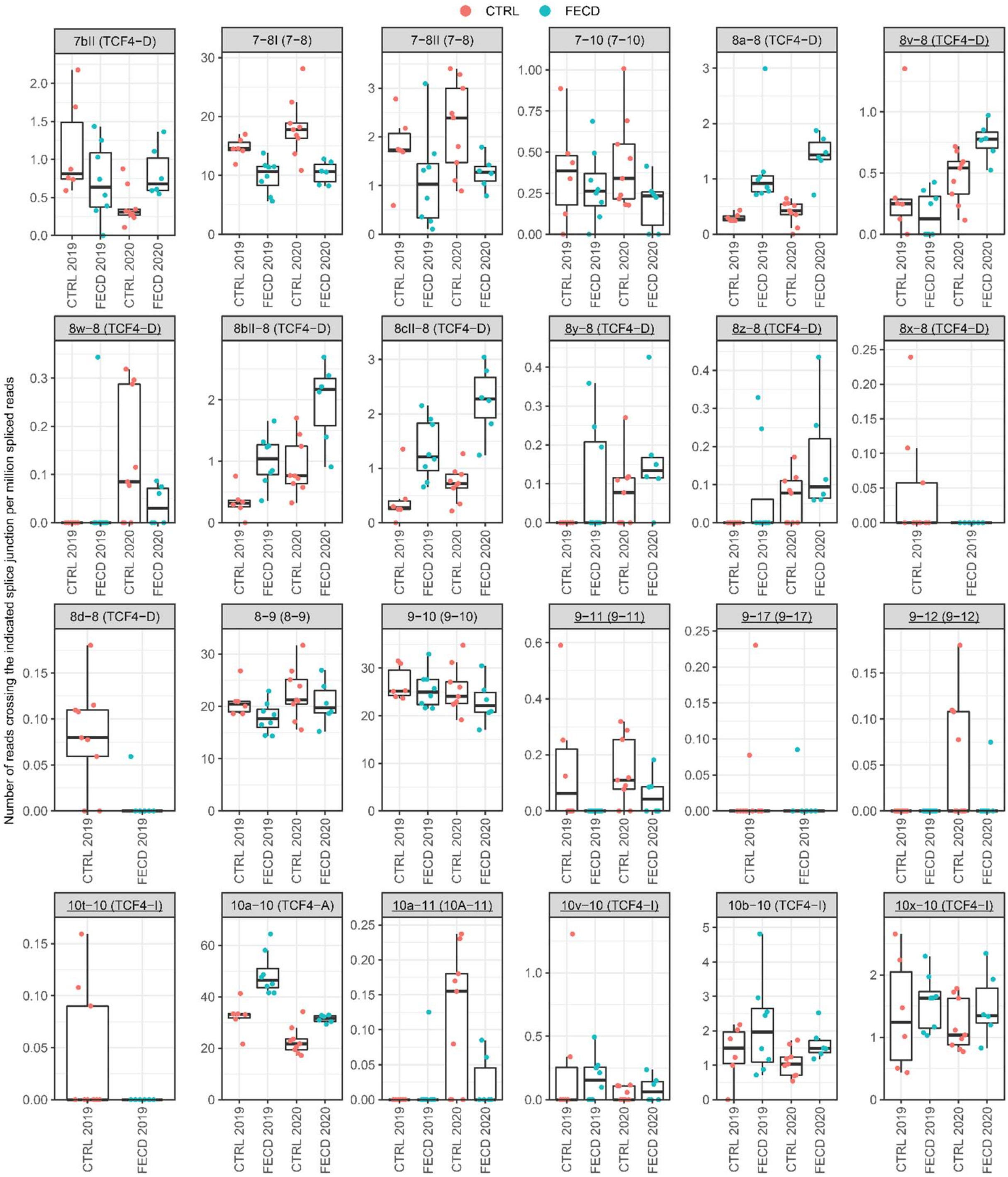

Figure continues on the next page.

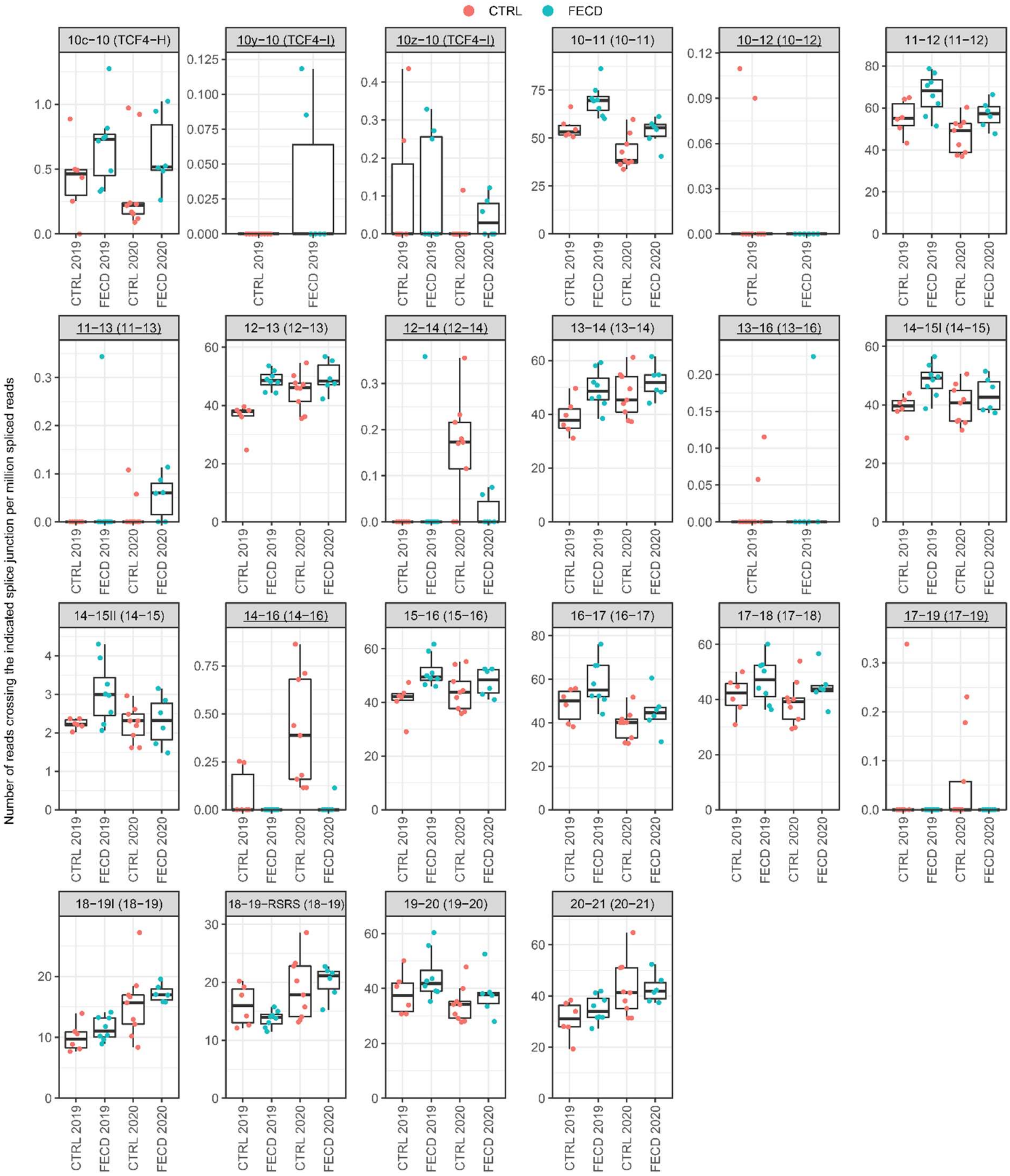

Supplementary Figure S2. Expression levels of *TCF4* isoforms and different splicing events in control groups and patients with FECD. Two independent FECD RNA-seq datasets – by Nikitina et al 2019[26] (named 2019 in the figure) and Chu et al 2020[27] (named 2020 in the figure) – were used to analyze the expression levels of *TCF4* exons in cornea. The expression levels of (A) TCF4 isoforms along with (B) 5' and internal exons were quantified using the number of reads (A) from transcripts encoding for the isoform (shown above the graphs) and (B) crossing the splice junction (shown above the graphs). Data was normalized with the total number of spliced reads and multiplied by million. The data is visualized as box plots – the hinges show 25% and 75% quartiles, the horizontal line shows the median value, the upper whisker extends from the hinge to the largest value no further than 1.5 * inter-quartile range from the hinge, the lower whisker extends from the hinge to the smallest value at most 1.5 * inter-quartile range of the hinge.

All data points are shown with dots. The 2019 dataset includes 6 controls and 8 FECD patients with an expanded *TCF4* CTG TNR, and the 2020 dataset includes 9 controls and 6 FECD patients with an expanded *TCF4* CTG TNR. Previously undescribed splicing events are underlined.

Supplementary Table S1. Primers used in this study.

Primers	Primer sequence and location	Primer location
**Primers for cloning**		
hTCF4_p4a_p4abc_s	CCCAAACACTTCAGCTAGAAACCAGTAGG	*TCF4* intron, between exon 3 and the CTG repeat
hTCF4_p4a_as	CTTCCCTGAGTCAGAGCCTGC	*TCF4* exon 4a
hTCF4_p4abc_as	GGAGGTGAAAACCTCTAAAAGAAACAAAG	*TCF4* exon 4
**Primers for sequencing**		
+53 ffLuc_as	TCGAGTGGGTAGAATGGCGCT	Downstream of the pGL4.15 cloning site
polyA_s	AGTGCAAGTGCAGGTGCCAGAACA	Upstream of the pGL4.15 cloning site
pST-Blue_as	CATTTAGGTGACACTATAGAAT	Downstream of the pSTBlue-1 cloning site
T7_s	TAATACGACTCACTATAGGG	Upstream of the pSTBlue-1 cloning site
**Primers for 5' RACE**		
hTCF4_exon4_as1	CCATTTTTCCCACTGCTCACAGGAG	Exon 4
hTCF4_exon4_as2	GTCCACTTGCCAAAGAAGTTGGTCC	Exon 4
hTCF4_exon4aI_as1	GGAGAAAGTGCAACAAGCAGAAAGG	Exon 4a before splice site I
hTCF4_exon4aI_as2	CCTGCAAAAAGCAAAGGAACGAATGG	Exon 4a before splice site I
hTCF4_exon4aII_as1	CTAATTGGTTTCCCTCTTCTTCGACG	Exon 4a between splice site I and II
hTCF4_exon4aIII_as1	CTCATTTCGTCTCTAACAGGAGAGC	Exon 4a between splice site II and III
hTCF4_exon4b_as1	CGTGGCAATGTCCATTTCCATCTCG	Exon 4b
hTCF4_exon4b_as2	GAGGTGAAAACATCTTGGCCATAAACG	Exon 4b
hTCF4_exon4c_as1	CTTGTGCCTTCAGAGCTCCTCAAAG	Exon 4c
hTCF4_exon4c_as2	ACAGTTGAAAAGAAGACACTTGTGCC	Exon 4c
**RT-PCR primers for exon 4a and 4b splicing and transcription analysis**		
hTCF4_4aI_s (1)	AATCCAAACCGCCTTCCAAGTG	*TCF4* intron – directly upstream of the CTG repeat
hTCF4_4aI_s (2)	CCTCTTCTAGACCTTCTTTTGGAG	Exon 4a – beginning, upstream of splice site 4aI
hTCF4_4aI_s (3)	CCCCTTTCTGCTTGTTGCACTTTC	Exon 4a – upstream of splice site 4aI

hTCF4_4aIII_s (1)	ACGTCGAAGAAGAGGGAAACC A	Exon 4a between splice sites I and II
hTCF4_4aIII_s (2)	GCTCTCCTGTTAGAGACGAAAT GAG	Exon 4a between splice sites II and III
hTCF4_4b_s	GAGATGGAAATGGACATTGCCA CG	Exon 4b
hTCF4_exon4_as 1	CCATTTTTCCCACTGCTCACAG GAG	Exon 4
hTCF4_exon4_as 2	GTCCACTTGCCAAAGAAGTTGG TCC	Exon 4

Supplementary Table S2. Scheme of primer usage in the 4 PCR steps of the 5'RACE analysis. GeneRacer 5' kit sense primer (Thermo Fisher Scientific) was combined with the TCF4-specific antisense primers as indicated.

Exon:	Exon 4a-I	Exon 4a-II	Exon 4a-III	Exon 4b	Exon 4c
Step 1	hTCF4_exon4_as2				
Step 2	hTCF4_exon4_as1				
Step 3	hTCF4_ exon4aI_as2	hTCF4_ exon4aII_as1	hTCF4_ exon4aIII_as1	hTCF4_ exon4b_as2	hTCF4_ exon4c_as2
Step 4	hTCF4_ exon4aI_as1	hTCF4_ exon4aI_as1	hTCF4_ exon4aI_as1	hTCF4_ exon4b_as1	hTCF4_ exon4c_as1

Supplementary Table S3. Sample information of the RNA-seq datasets used for bioinformatical analysis. (A, B) Sample ID, gender, age and the CTG TNR length in *TCF4* is shown for the patients sequenced in Nikitina et al 2019 (A) and Chu et al 2020 (B).

A

Sample ID	Gender	Age	*TCF4* CTG TNR length
C_201	F	47	14, 18
C_203	M	63	11, 18
C_205	F	54	11, 17
C_206	F	65	11, 15
C_207	F	64	11, 17
C_208	M	61	17, 17
Dfu_201	F	70	12, 156
Dfu_202	M	63	72, 72
Dfu_203	F	57	72, 72
Dfu_205	F	64	32, 127
Dfu_207	F	79	11, 87
Dfu_212	F	72	14, 44
Dfu_213	M	74	14, 104
Dfu_215	F	70	21, 114

B

Sample ID	Gender	Age	*TCF4* CTG TNR length
Control_0	F	74	16, 39
Control_1	F	72	16, 25
Control_2	M	51	16, 17
Control_3	M	68	18, 25
Control_4	M	68	18, 25
Control_5	M	71	12, 18
Control_6	F	61	13, 19
Control_7	M	74	13, 18
Control_8	F	43	12, 18
FECD_REP_0	F	67	14, 87
FECD_REP_1	M	66	15, 150
FECD_REP_2	F	68	15, 77
FECD_REP_3	F	58	16, >100
FECD_REP_4	F	69	15, >100
FECD_REP_5	F	71	19, 130
